# Internet-Based Health Information–Seeking Behavior of Students Aged 12 to 14 Years: Mixed Methods Study

**DOI:** 10.2196/16281

**Published:** 2020-05-26

**Authors:** Emanuel Maitz, Katharina Maitz, Gerald Sendlhofer, Christina Wolfsberger, Selma Mautner, Lars-Peter Kamolz, Barbara Gasteiger-Klicpera

**Affiliations:** 1 Division of Plastic, Aesthetic and Reconstructive Surgery Department of Surgery Medical University of Graz Graz Austria; 2 Inclusive Education Unit Institute of Professional Development in Education University of Graz Graz Austria; 3 Division of Neonatology Department of Pediatrics and Adolescent Medicine Medical University of Graz Graz Austria

**Keywords:** internet-based health information–seeking behavior, eHealth literacy, children and adolescents, mixed methods study

## Abstract

**Background:**

Many children and adolescents are surrounded by smartphones, tablets, and computers and know how to search the internet for almost any information. However, very few of them know how to select proper information from reliable sources. This can become a problem when health issues are concerned, where it is vital to identify incorrect or misleading information. The competence to critically evaluate digital information on health issues is of increasing importance for adolescents.

**Objective:**

The aim of this study was to assess how children and adolescents rate their internet-based health literacy and how their actual literacy differs from their ratings. In addition, there was a question on how their search performance is related to their self-efficacy. To evaluate these questions, a criteria-based analysis of the quality of the websites they visited was performed. Finally, the possibility to increase their internet-based health literacy in a 3-day workshop was explored.

**Methods:**

A workshop with a focus on health literacy was attended by 14 children and adolescents in an Austrian secondary school. After prior assessments (Culture Fair Intelligence Test, revised German version; Reading Speed and Reading Comprehension Test for Grades 6 to 12, German; electronic health literacy scale [eHEALS]; and General Self-Efficacy Scale, Reversed Version, German), the students were asked to perform an internet-based search on a health-related issue. Browser histories and screenshots of all internet searches were gathered, clustered, and analyzed. After the workshop, the health literacy of the students was assessed again by using the eHEALS.

**Results:**

The 14 students opened a total of 85 homepages, but only eight of these homepages were rated as good or fair by two experts (independent rating) based on specific criteria. The analysis showed that the students judged their own internet-based health literacy much higher than the actual value, and students who had rated themselves better did not visit websites of high quality. Internet-based health literacy correlated significantly with the self-efficacy of the students (r_s_=0.794, *P*=.002).

**Conclusions:**

Our study showed that it is possible to draw the attention of students to critical aspects of internet search and to slightly improve their search competence in a workshop. Targeted improvement of health literacy is urgently required, and students need special instruction for this purpose. Further investigations in this area with larger sets of data, which could be feasible with the help of a computer program, are urgently needed.

## Introduction

At present, a huge amount of information is accessible on the internet at almost any time and any place. In the United Kingdom, in 2015, 82.8% of adolescents (mean age 13.67 years) used the internet between 1 and 4 hours a day [1]. In the United States, in 2015, 92% of teenagers (aged 13-17 years) used the internet daily, with only 8% of them using the internet once a week or less [2]. In Austria, in 2019, 94% of Austrian youth (aged 11-14 years) had access to the internet at home and spent on average 94 minutes a day using the internet, and 62% of them searched for information using the internet [3]. This is consistent with the finding that 64% of all young people (aged 16-29 years) in the European Union searched for information using the internet in 2015 [4].

The information seeking process usually starts with a need for information [5], which is often satisfied by using search engines (eg, Google) on the internet [6]. Search engines usually require the user to express an information need by formulating a search query. Search queries submitted to a search engine can be quite different, ranging from keywords to whole questions. A recent study showed that children and young adolescents (aged 11-13 years) mainly formulate phrase- and question-like queries rather than keywords when searching for information regarding a particular task [7]. Moreover, young students were more likely to use questions, and the use of keyword queries increased with an increase in the grade level [7]. In this context, a previous study showed that using question-like queries can be advantageous, at least for unexperienced users like young children who have great difficulties in formulating precise and comprehensive keyword queries [8].

Another challenge for children and adolescents who use search engines is the ranking of search results by search engines, with the placement of paid advertisements first, rather than more reliable sources. Children and young adolescents tend to open the top-ranked results, dismissing lower-ranked results [9,10]. In addition, after opening one of the top results, they frequently continue their search only if they are not satisfied with the obtained information. If the first page clicked provides an answer to their question, they complete their internet-based search without evaluating the source [6,11]. However, evaluating the source can help readers assess the reliability and quality of the provided information [12,13]. Knowledge about the person or organization publishing the information can help to recognize the intention of publication (whether it is professional or for sales or marketing). The publication date or the last time a webpage was updated can help to estimate if the information provided is still relevant or is outdated [14,15]. Therefore, readers should evaluate information from authors or webpages they identify as being appropriate [12,14,16].

Children and adolescents in primary and secondary schools usually do not evaluate the sources and information they read on the internet [14,17-19]. They often only evaluate the length of text or the design of a webpage [20]. When middle school students (aged 10-15 years) are asked to evaluate an internet source, it is usually very difficult for them to determine which person or organization authored the information and to assess the actual expertise of the author [11]. Although ninth grade students have a basic idea of the concept of evaluation criteria, they often have difficulties in applying them to information on the internet [19].

Query formulation and source evaluation skills are especially important in health-related searches, because the majority of internet users have searched for health information on the internet at least once [21-23]. Besides health professionals (eg, doctors and nurses) and family or friends, the internet is among the first three choices for most people who have health-related questions [22]. In the European Union, the proportion of people aged 16 to 74 years seeking health-related information on the internet increased from 24% to 51% between 2007 and 2017 [24]. A survey in 2016 showed that 84% of teenagers in the United States (aged 13-18 years) had obtained health information on the internet previously, with 38% searching the internet for health information once a year and 24% searching at least monthly or more frequently [25]. Search engines (eg, Google) are usually used to search for health information on the internet, whereas health portals are rarely used [6,25].

Although the internet can be a very powerful source of health-related information, many experts criticize its trustworthiness. The quality of internet-based health information is consistently rated as problematic [26-29]. Commercial sites, discussion lists, online support groups, and newsgroups frequently provide poor quality or even false health information. In many cases, this information does not meet safety and quality standards. It rather promotes nonevidence-based treatments, provides advice to generally avoid drug intake, or omits possible drug interactions. Furthermore, the content of such webpages is seldom updated [27].

Children and adolescents are rarely aware of these shortcomings of health information on the internet and are especially easily influenced by false or biased information [14,17-19]. Despite the obvious need for educational interventions in schools targeting competence regarding internet-based health information and systematic source evaluation, the respective skills are not part of the national curriculum in most education systems [20,30]. However, in several countries, including Austria, health education is part of the core curriculum [31,32]. In the last decade, the perception of what health education should contain has slowly changed, drawing the attention of policy makers to the concept of health literacy as a complement to health promotion and disease prevention.

Health literacy is defined by the US Department of Health and Human Services as follows: “The degree to which individuals have the capacity to obtain, process, and understand basic health information and services needed to make appropriate health decisions.” [33]. Health literacy means the knowledge and competence of finding and understanding health-related information, as well as dealing with that information. For appropriate handling of health-related internet-based information, a high level of health literacy is essential. Health information–seeking skills (finding information) and source evaluation skills (appraising information to make appropriate decisions) can be considered strongly related to health literacy. In addition to information seeking skills and source evaluation skills, reading literacy (understanding information) and self-efficacy are important influencing factors for health literacy [11]. The concept of self-efficacy was discovered and described by the sociocognitive action theorist Albert Bandura [34-36] and means “own trust in one’s own ability.” Self-efficacy regarding information seeking and understanding is considered important to start the information seeking process in the first place [11] and supports a person’s inclination to make health-related decisions.

Despite an increased awareness of the importance of health literacy in general and the teaching of health literacy–relevant skills, evidence-based intervention programs to improve health literacy in children and adolescents are rare. Often teachers are not familiar with this concept or do not know how to address health literacy in their lessons. One of the main reasons is the lack of valid data regarding the current internet health information–seeking behavior of children and adolescents, especially in Europe (no data available in Austria).

Therefore, the aim of this mixed methods study was to assess the internet-based health information–seeking behavior of secondary school students (aged 12-14 years) in Austria and focus on the quality of information sources used by this age group. The first goal was to find out how competent children and adolescents are when dealing with health-related information on the internet. Additionally, the strategies they used when searching the internet were observed. Finally, we aimed to find out if a 3-day training on interned-based research competence and reading strategies could enhance the students’ health literacy. The following research questions were addressed: (1) How did the students rate their internet-based health literacy? (2) Which websites did they visit to access health-related information and what was the quality of the visited websites (before the students received any training)? (3) How does the students’ perceived internet-based health literacy relate to the actual quality of their internet search and the selected sources and how accurate is their assumption of their own health literacy? (4) Is there a relationship between the change process of the students’ internet-based health literacy (during a workshop that aimed at improving internet health information–seeking behavior and source evaluation) and their general self-efficacy?

## Methods

### Set-Up and Participants

From March 20 to 22, 2018, we held a workshop that focused on health literacy in Austrian lower secondary students. The workshop was held by one medical student and one education researcher on 3 consecutive days and comprised 12 class hours in total. It took place in the computer laboratory of the school. As our understanding of health literacy includes internet-based information literacy and reading literacy, we addressed these topics explicitly. The participants of the workshop were between 12 and 14 years old, and all of them went to the same third-grade class of a secondary school in Austria. A total of 14 students (eight girls and six boys) participated, and all of them were born in Austria with German as their native language. The parents and the students themselves provided written consent for participation in the workshop and the study.

### Instruments

The control variables *general cognitive abilities* and *reading competence* were assessed prior to the workshop using the Culture Fair Intelligence Test, revised German version [37] and the Reading Speed and Reading Comprehension Test for Grades 6 to 12, German [38], respectively. In addition, we asked students about their habits of internet use.

We used the electronic health (eHealth) literacy scale (eHEALS), which has eight statements (Table 1) and an internal consistency of α=.88, to assess the students’ internet-related health literacy (pre- and postintervention; on the first day of the workshop and 2 weeks after the workshop) [39]. The statements were scored on a five-point Likert scale with response options ranging from *strongly agree* to *strongly disagree*. For further computations, we reversed the polarity of the scale so that a higher mean score indicated a higher eHealth literacy.

Further, we assessed the general self-efficacy of the participants with the General Self-Efficacy Scale, Reversed Version, German [40], which consists of 10 statements, with four response options for each statement ranging from *strongly disagree* to *strongly agree*. A higher mean score indicated a higher general self-efficacy with sufficient internal consistency between α=.78 and α=.79 [41].

### Procedure

At the beginning of the workshop, we asked the students to perform internet-based research on a health-related issue that was described in short narrative text in the fashion of a vignette with a research task. The topics addressed in the vignette were defined by the researchers and the class teacher. We divided the students into two groups (for didactical reasons, it was easier to work with smaller groups), and each group received one of two vignettes (vignette 1 and vignette 2).

Vignette 1 was as follows: “A friend of yours, his name is Klaus, meets with you to discuss something. So far, he has not talked to anyone about it, but now he wants to ask you for some advice. Klaus tells you that since his childhood, he has been bothered about the appearance of his nose, which he finds way too big and too crooked. He is considering having his nose corrected surgically. Now, he would like to know your opinion. You tell him that you would like to give him some advice, but first you want to know more about rhinoplasty. Try to inform yourself using the internet and make a profound decision about what advice you will give Klaus.”

Vignette 2 was as follows: “Imagine you have a friend named Claudia. Claudia has a large dark mole on her left cheek since birth. Making matters worse, this mole has dark bristly hair. Claudia tells you that this spot on her cheek has been bothering her since she can remember. In addition, she is afraid that the mole could become dangerous, as some moles do. Now, she asks you if you think she should get rid of the mole. You explain that you would like to give her some advice, but first want to know more about the condition. Try to inform yourself using the internet and make a profound decision about what advice you will give Claudia.”

Before and during their search the students did not receive any input on how to search efficiently on the internet or how to evaluate sources. We instructed them to take screenshots of all their Google searches (the students were most familiar with this search engine) and of all webpages they opened. These data were used to determine how they performed their internet-based search and to discuss their search behavior and their results with them later.

Following their search, the students wrote down the advice they would give to their friend (Klaus or Claudia) and why they would give that advice. Thereafter, we discussed the sources they chose and why they chose them. After this first day, we gathered the browser histories of all students and the screenshots they had taken during their searches, as well as the notes they had made on what advice they would have given their friend. On the second and third days of the workshop, the students received input and performed tasks regarding proper search, reading strategies, source evaluation, and reliable internet sources on health issues.

### Data Analysis

We clustered and evaluated the browser history and screenshots the students made on the first day of the workshop in order to analyze their internet search behavior. We assessed all visited websites following the classification system of Pérez et al [42] who suggested to distinguish four stages of information quality according to the following (independently rated) criteria: media quality (eg, *A forum versus a website of the Ministry of Health*), author’s motivation (eg, *A surgeon with a private practice* versus *a researcher for evidence-based medicine*), and author’s position (eg, *A boulevard journalist* versus *a psychologist with specialization in body perception disorders*). Two researchers rated all visited websites independently and subsequently compared and discussed their ratings. There was consensus regarding all websites, except one. For this website, a third researcher was consulted, and eventually, we were able to assign all of the websites to one of the following categories: (1) *good* (3 points; all three dimensions are reliable); (2) *fair* (2 points; two of the three dimensions are reliable); (3) *poor* (1 point; one of the three dimensions is reliable); and (4) *bad* (0 points; all of the three dimensions are unreliable).

Descriptive statistical analyses were performed using IBM SPSS Statistics 25 (IBM Corp, Armonk, New York, USA) [43].

## Results

### Participant Findings

All participating students had average IQ values (mean 99.77, SD 8.66). In relation to an age-equivalent norm sample, the average performance was within the norm for this cohort in reading comprehension (mean T value 50.07, SD 8.13), and the students were slightly faster than the norm sample in reading speed (mean T value 54.07, SD 5.62). There was a noticeable variation in reading competence among the students; however, reading competence had no relevant associations with seeking behavior-related variables, the eHEALS score, or self-efficacy. Furthermore, on comparing the reading comprehension score with the search behavior of the students, no systematic patterns were identified. A higher reading competence did not lead to a more sophisticated search or better source evaluation.

All students had access to web-enabled devices, such as tablets, smartphones, and computers, at home and reported searching for information on the internet at least several times a month.

### Students’ Self-Assessment of Their Internet-Based Health Literacy

The self-assessment of internet-based health literacy before the workshop (Table 1) resulted in a rather high mean overall eHEALS score (range 1-5) of 3.5 (SD 0.7). All students indicated that they knew how to find helpful health resources on the internet (Table 1, Q1). In contrast, their answers regarding the availability of health resources on the internet suggested that they did not know which health resources were available or they had little experience with internet-based health resources so far. A detailed inspection of the eHEALS scores (Table 2) showed a high diversity within the scores of the students. The average scores ranged from below the scale mean (student 1) to nearly the highest possible value (student 12). Furthermore, there were hardly any differences between female and male students.

**Table 1:** Electronic health literacy scale questions and their scores before the workshop.

eHEALS^a^ questions	Results before the workshop	
	Participants, n	Score, median (IQR)
Q1: I know how to find helpful health resources on the internet	10	5 (4-5)
Q2: I know how to use the internet to answer my health questions	13	4 (4-5)
Q3: I know what health resources are available on the internet	13	2 (1.5-3)
Q4: I know where to find helpful health resources on the internet	14	3 (1-4)
Q5: I know how to use the health information I find on the internet to help myself	13	4 (4-5)
Q6: I have the skills I need to evaluate the health resources I find on the internet	14	3.5 (2-4)
Q7: I can tell high quality from low quality health resources on the internet	12	3.5 (2-4)
Q8: I feel confident in using information from the internet to make health decisions	13	4 (3.5-4)

^a^eHEALS: electronic health literacy scale.

**Table 2:** Electronic health literacy scale scores of the students before the workshop.

Student number (sex)	Mean eHEALS^a^ score
1 (F)	2.29
2 (F)	2.63
3 (F)	2.71
4 (M)	3.29
5 (F)	3.38
6 (M)	3.5
7 (M)	3.63
8 (F)	3.75
9 (F)	3.88
10 (F)	4.38
11 (F)	4.38
12 (M)	4.5
13 (M)	N/A^b^
14 (M)	N/A

^a^eHEALS: electronic health literacy scale.

^b^N/A: not applicable; data regarding the eHEALS score are not available.

### Quality of the Visited Websites

All participants showed similar internet-based information–seeking behavior no matter how competent they rated themselves in the eHEALS questionnaire.

The participating 14 students entered a total of 44 search requests into Google and visited a total of 85 webpages for the task. Among these webpages, 2 (2%) were rated as *good*, 6 (7%) as *fair*, 37 (44%) as *poor*, and 40 (47%) as *bad* by independent raters. Hence, more than 90% of the webpages were classified as *poor* or *bad*. Almost half of the webpages visited were homepages of doctors with obvious commercial backgrounds. These were paid advertisements on the top of the search results list and were labelled accordingly. Only one student visited a webpage of a university providing evidence-based medical information, and another student visited a high-quality medical internet-based encyclopedia. Both webpages were rated as *good* (Table 3).

**Table 3:** Visited webpages and ratings.

Webpages	Rating (score)^a^	Total visits, n
A medical specialist portal with a medical internet-based encyclopedia for health professionals	Good (3)	1
Evidence-based medical sites without a direct commercial endeavor	Good (3)	1
Video sharing service	Fair (2)	4
An internet-based encyclopedia after the Wiki-Principle	Fair (2)	2
Homepages of doctors with economic backgrounds (paid ads)	Poor (1)	37
Internet-based magazines	Bad (0)	15
Forums	Bad (0)	8
An internet-based tutorial and how-to page	Bad (0)	7
Economically oriented websites (consumer products)	Bad (0)	4
Alternative medicine or pseudomedically oriented pages	Bad (0)	6

^a^Rating was according to the classification scheme of Pérez et al [42].

### Perceived Internet-Based Health Literacy Compared With the Actual Quality of Internet Searches and Selected Sources

The mean number of Google searches with unique queries initiated by the participants was 3.14 (SD 1.99). The number of opened websites ranged from 2 to 16 (Table 4). We found no significant correlation (*r_s_*=0.158, *P*=.59) between the self-assessed internet-based health literacy of the participants and the actual quality of their internet searches. The results of three students were particularly noticeable. Student 2 rated internet-based health literacy relatively low but performed six Google searches and visited eight websites with an average quality of 1.0. In contrast, student 12 had the highest eHEALS score and visited 16 websites, but the quality of the websites was *poor* or *bad*. Student 9 had the highest average website quality (1.5), and this student’s eHEALS score was relatively high.

**Table 4:** Electronic health literacy scale scores, searches, and search qualities of the students.

Student number (sex)	eHEALS^a^ score before the workshop	Number of Google searches	Number of visited websites	Average quality of the websites	Rating (score)^b^, number of websites			
					Good (3)	Fair (2)	Poor (1)	Bad (0)
1 (F)	2.29	3	8	0.13	—	—	1	7
2 (F)	2.63	6	8	1	—	2	4	2
3 (F)	2.71	3	5	0.4	—	—	2	3
4 (M)	3.29	1	3	1	—	—	3	1
5 (F)	3.38	3	7	0.29	—	—	2	5
6 (M)	3.5	3	5	1	—	1	3	1
7 (M)	3.63	2	5	0.4	—	—	2	3
8 (F)	3.75	1	4	0.5	—	—	2	2
9 (F)	3.88	1	4	1.5	1	—	3	—
10 (F)	4.38	1	2	0	—	—	—	2
11 (F)	4.38	3	5	1,2	—	1	4	—
12 (M)	4.5	7	16	0.44	—	—	7	9
13 (M)	N/A^c^	4	7	0.71	1	1	1	4
14 (M)	N/A	6	6	0.67	—	1	3	2

^a^eHEALS: electronic health literacy scale.

^b^Rating was according to the classification scheme of Pérez et al [42].

^c^N/A: not applicable; data regarding the eHEALS score are not available.

### Relationship Between the Change Process of the Students’ Internet-Based Health Literacy and Their General Self-Efficacy

Regarding the difference in eHEALS scores before and after the workshop, we found that the mean eHEALS score increased slightly from 3.5 (SD 0.7) before the workshop to 4.0 (SD 0.5) after the workshop. However, not all students had a higher eHEALS score after the workshop. Those students who estimated their internet-based health literacy as particularly high before the workshop had lower ratings after the workshop and vice versa. We also found a significant correlation (Spearman rank correlation coefficient) between the eHEALS score before the workshop and the students’ general self-efficacy (*r_s_*=0.794, *P*=.002). Participants with a high self-efficacy rated themselves higher in the eHEALS as compared to those with lower self-efficacy. In contrast, participants with high self-efficacy rated their internet-based health literacy lower and those with low self-efficacy rated their internet-based health literacy higher after the workshop than before the workshop (Table 5).

After the workshop, on average, the participants rated themselves better for eHEALS questions Q2, Q3, Q4, Q6, and Q7, but worse for questions Q1, Q5, and Q8. In particular, the effects of the workshop were noticeable for Q1 (“I know how to find helpful health resources on the internet”) and Q3 (“I know what health resources are available on the internet”). The score for Q1 was high before the workshop and slightly lower after the workshop. This question was apparently particularly difficult for the students to answer before the workshop, as only 10 of the 14 students provided an answer. The score for Q3 changed in the opposite direction with an increase in the median (Table 6).

**Table 5:** Difference in electronic health literacy scale scores before and after the workshop and self-efficacy.

Student number	eHEALS^a^ score before the workshop	eHEALS score after the workshop	Difference in the eHEALS score	Self-efficacy score
2	2.63	4.17	1.54	2.33
4	3.29	4.75	1.46	2.3
5	3.38	4.14	0.77	1.78
9	3.88	4	0.13	2.6
7	3.63	3.75	0.13	2.78
6	3.5	3.5	0	2.3
10	4.38	3.75	−0.13	3.8
12	4.5	4.13	−0.38	3.14
11	4.38	4.25	−0.63	3.78
8	3.75	3	−0.75	3.5

^a^eHEALS: electronic health literacy scale.

**Table 6:** Electronic health literacy scale questions and their median scores before and after the workshop.

eHEALS^a^ questions	Results before the workshop		Results after the workshop		
	Participants, n	Score, median (IQR)		Participants, n	Score, median (IQR)
Q1: I know how to find helpful health resources on the internet	10	5 (4-5)		12	4 (4-5)
Q2: I know how to use the internet to answer my health questions	13	4 (4-5)		12	5 (4.25-5)
Q3: I know what health resources are available on the internet	13	2 (1.5-3)		12	4 (2.25-4)
Q4: I know where to find helpful health resources on the internet	14	3 (1-4)		12	4 (3.25-4.75)
Q5: I know how to use the health information I find on the internet to help myself	13	4 (4-5)		12	4 (3-4.75)
Q6: I have the skills I need to evaluate the health resources I find on the internet	14	3.5 (2-4)		12	4 (4-5)
Q7: I can tell high quality from low quality health resources on the internet	12	3.5 (2-4)		10	4 (3-5)
Q8: I feel confident in using information from the internet to make health decisions	13	4 (3.5-4)		11	3 (2-4)

^a^eHEALS: electronic health literacy scale.

### Students’ Statements Regarding Their Learning Experience During the Workshop

At the final interview after the workshop, the students acknowledged the usefulness of the workshop and the importance of critically reviewing information on the internet. For example, one student described the main insights as follows:

The search results […] there are mostly ads on the first page and that they only present positive things on their webpages. And only positive things, therefore no negative things. Therefore, you should use general pages that have no special intention, where you can look for advantages and disadvantages (of a treatment).

Student #12, male, 14 years

Another student pointed out a sense of achievement as follows:

And later we also searched and found many sources where you can say “yes ok now I have found something useful” and now I can use (the information) to help someone.

Student #8, female, 13 years

Results from a brief evaluation questionnaire filled out immediately after the workshop by the students revealed that they were overall satisfied with the workshop and with what they had learned. They described the workshop as “beneficial,” “fun,” and “cool.”

## Discussion

### Principal Findings

The purpose of this study was to obtain initial insights into the internet-based health information–seeking behavior of children and adolescents aged between 12 and 14 years. We used an explorative approach to examine the internet-based health information–seeking behavior of the students during an interactive workshop. With this approach, we wanted to gain important insights into a previously sparsely investigated yet crucial subarea of health literacy.

At the beginning of the workshop, all students stated that they actually had limited knowledge about what kind of health information was available on the internet and where to find it. However, they thought that it would be easy to find health-related information on the internet and to evaluate this information.

We found no correlation between this self-assessment of the students and their actual seeking behavior. For example, there was one student who rated internet-based health literacy particularly low but performed a relatively good internet-based search. However, the overall internet-based seeking behavior was rather superficial for nearly all students, regardless of their mostly positive self-view. The first three results presented by the search engine were mainly visited, and the students did not consider whether the information was a paid advertisement. Paid advertisements were mainly by doctors, internet-based magazines, and forums, which were rated as *poor* or *bad* by experts. Among all the visited websites, only two were rated as *good* and the vast majority of websites were rated as either *poor* or *bad*. These results are in line with the findings of previous research [11] showing that youth tend to trust webpages merely because the owner is a doctor or has a similar profession, regardless of the intention (eg, selling products) of the authoring person.

The eHEALS score increased slightly after the workshop, indicating that the students had gained more confidence and competence in the areas of finding and evaluating internet-based health information. After the workshop, the participants were more skeptical about the quality of information on the internet and they were less trusting. This suggests an increased awareness of the students for the large amount of poor-quality health information on the internet.

The results of our study clearly support the findings of previous studies [11,21,44], indicating that students are not used to evaluating internet-based health information critically with objective criteria. This applies to internet users in general, as stated by Sun et al [12], and students in particular [11,21]. To understand the underlying mechanisms of webpages, students need information and training. This training should be given at a young age before they enter the critical phase of adolescence. As youth often develop risky behaviors at this age (eg, risky sexual behavior and smoking or substance abuse), they need to be prepared for critical health issues. Otherwise, the full effects and consequences of such behaviors will become clear in the later stages of life [45].

It is unlikely that the internet’s importance as a source of health information will decrease or that nonreliable health-related information will vanish from the internet. There is an urgent need to teach children and adolescents how to properly handle internet-based health information. This could be supported by implementing health literacy in the regular curriculum and developing training programs that foster appropriate and critical internet-based health information seeking. The benefits of such measures would be substantial, not least because increased health literacy can reduce costs for health care systems when individuals rely on high-quality information to make health decisions [46-48].

### Limitations

The most important limitation of the study is the small sample size. We cannot assume that the 14 students represent all other Austrian adolescents of similar age. Nevertheless, the results give rise to concerns because despite the low number of students in our evaluation, we expect the results to be reflected in a larger population of students at this age. To prove this assumption, further investigations in this area with larger samples are urgently needed. Additionally, to validate the results, a possible approach could be the application of a computer software that is being newly developed for this purpose.

The study was also limited by the use of the eHEALS, which is based on self-assessment and thus is not an objective instrument for measuring health literacy. The eHEALS scores may be confounded with other competencies, such as reading comprehension and general cognitive abilities. We tried to clarify this aspect, but it remains problematic, because this also applies to the concept of health literacy as a whole. Finally, we noticed a relevant correlation between the eHEALS score before the workshop and the students’ general self-efficacy, which might be a confounding factor in measuring health literacy.

### Conclusion

The results of this study shed light on the very critical aspects of health literacy and information–seeking behavior. We showed that it is possible to draw the attention of students on the critical aspects of internet search, as well as to slightly improve their competence through a workshop. Although it is very difficult to promote health literacy over a limited time period of 3 days, we could raise awareness of this important topic among students and teachers. Finally, the results highlight that targeted promotion of health literacy, as well as further research is urgently needed, especially among children and adolescents.

## Abbreviations

eHEALS: electronic health literacy scale
eHealth: electronic health
